# Iranian nurses' constraint for research utilization

**DOI:** 10.1186/1472-6955-8-9

**Published:** 2009-09-13

**Authors:** Mahvash Salsali, Neda Mehrdad

**Affiliations:** 1 Nursing Faculty, Tehran university of Medical Sciences, Tehran, Iran

## Abstract

**Background:**

This paper identifies the views of Iranian clinical nurses regarding the utilization of nursing research in practice. There is a need to understand what restricts Iranian clinical nurses to use research findings. The aim of this study was to identify practicing nurses' view of aspects which they perceived constrain them from research utilization that summarizes and uses research findings to address a nursing practice problem.

**Methods:**

Data were collected during 6 months by means of face-to face interviews follow by one focus group. Analysis was undertaken using a qualitative content analysis.

**Results:**

Findings disclosed some key themes perceived by nurses to restrict them to use research findings: level of support require to be research active, to be research minded, the extent of nurses knowledge and skills about research and research utilization, level of educational preparation relating to using research, administration and executive challenges in clinical setting, and theory-practice gap.

**Conclusion:**

This study identifies constraints that require to be overcome for clinical nurses to actively get involved in research utilization. In this study nurses were generally interested to use research findings. However they felt restricted because of lack of time, lack of peer and manager support and limited knowledge and skills of the research process. This study also confirms that research utilization and the change to research nursing practice are complex issues which require both organizational and educational efforts.

## Background

Seventy thousands nursing staff have been employed to provide nursing care in Iranian hospitals. Nursing is the largest healthcare profession in Iran, and they are responsible for the care they provide for their patients. If the available research is utilized properly, the nurses can be held accountable for their action in daily practice. Iranian nurses have been criticized for poor quality of patient care and informed by the healthcare system that nursing practice should be carried out based on international standards to improve the quality of patient care[1].

Research utilization is a critical way to strengthen the discipline and practice of nursing.

Improving research utilization will provide nurses with the 'golden opportunity' to meet the patients' caring demands with the appropriate positive responses.

This research-based practice will lead to the improvement of the nursing profession.

Research utilization is now part of the nurses' professional role and responsibility, which has been intertwined with concepts of efficiency, effectiveness and quality improvement in health care [2,3]. In a profession such nursing, it is important that nurses demonstrate that their practice is effective, efficient and worthwhile [4], and more likely to be appropriate and justifiable.

In spite of the increase in the quantity and quality of nursing research and included research content in current nursing curricula, research knowledge among nurses and use of research in practice remains poor [5]. In addition, utilization of research evidence by nurses in clinical settings is still not perceived favorably or used proactively by the majority of nurses [6] and it is not parallel with the increase in research output. Research utilization is of peripheral importance to nursing staff.

Nevertheless, in achieving a health care service of high quality, the implementation of research findings is an issue of important concern for the nursing profession [7]. So it is time to further consider another part of the research spectrum, namely research utilization in nursing practice.

The notion that research-based knowledge is necessary to improve clinical practice is not new [6]. However, research utilization is a new paradigm in Iranian nursing and nursing in Iran is still a long way from undertaking research that influences practice [8]. In Iran, as in most Middle East countries, increasing cost limitations, a push for quality of clinical care, and patient-centered care are some of the forces that require health care to be based on scientific research not only in Iran and developing countries but also around the world [1]. Iranian nurses need to use sound research evidence in practice. The health care system is searching for programs and strategies to establish research-based nursing practice. Thus, an important area for using research findings is to obtain nurses' perspectives on factors that constrain research utilization in nursing practice. It could be the first essential step for nursing administrators and educators, when designing programs for getting research utilization into education and practice. To this end a qualitative content analysis was conducted to identify practicing nurses' view of aspects that they perceived restricted them from research utilization.

### Barriers of research utilization

The body of literature is vast. Throughout the last two decades the barriers of research utilization in clinical practice have been repeatedly stressed. There are several reasons why nurses do not use research findings, and multiple studies have revealed barriers of research utilization [9-12]. One influential study was undertaken by Funk et al (1991, 1995) who developed and validated the BARRIERS Scale [13,14]. It has been used in several published studies to elicit nurses' perception regarding barriers to research utilization in the UK [10] the USA [9], Ireland [15], Norway [16] Greece [17] and Iran [1]. Most studies have been conducted using survey approaches and rely on self reported utilization behavior [14,18,19]. For example Mehrdad et al (2008 a) used a descriptive design to identify barriers and facilitators of research utilization in nursing practice from the Iranian nurses self reports [1].

The value of self-report studies per se may be questioned in relation to the veracity of the results, due to the possibility of inflated reports of research utilization by those responding. This cannot be relied upon to present the most valid outcome [20]. Moreover, these types of studies tend not to concede the interaction of the individual with contextual factors, which may be an important factor in successful implementation [21].

The employment of qualitative research methods will contribute further to our knowledge about barriers of research utilization by nurses by allowing deeper exploration of experiences, perception and issues faced by nurses in the utilization of research in their practice [11]. Such an approach also allows us to draw upon participants' attitudes, experiences, and reactions in a way that would not be feasible using self-report studies [22]. Although the studies are carried out in several countries, the factors inhibiting research utilization have not been well explored in the Iranian national health system and particularly within the discipline of nursing in Iran since the context is different. It is an urgent need to develop nursing knowledge based on the health of the global community. We need to understand constraints of research utilization in several countries. We need to integrate collaborative international research in the ongoing work of scientists in leading nursing academies throughout the world. And we need to pay attention to the freedom of thought and action which is a fundamental value among university members. Therefore, it is time to identify Iranian clinical nurses' views on issues that hinder research utilization in nursing practice.

## Methods

A qualitative approach has been used to emerge a much richer picture and to explore nurses' understanding of what constrains Iranian clinical nurses from using the research.

To facilitate access to informants' perceived data, all male and female staff nurses with at least one year of nursing experience, have minimum a bachelor's degree, who work full-time, and provided nursing care in different wards of large general and specialty hospitals covered by Tehran University of Medical Sciences were considered as potential participants. The semi-structured interviews were conducted with 15 participants based on the aims of the study. It was a conversation between the participant and investigator.

Each interview was planned. An interview guide was organized to make sure that certain information about research utilization was collected.

The researchers used snowball and purposeful sampling to access main information about research utilization in clinical nursing practice until saturation had been achieved.

The participants are considered typical of the study population and capable of answering the research questions [23].

The study was followed by a focus group to collect ideas and feedback. The group members influence each other by responding to ideas and comments in the discussion.

The focus group was held by seven other participants from same hospitals in the clinical ward and lasted more than one hour. Each face to face interview lasted approximately 1 hour and took place in a setting of the participant's choice. Interviews and discussion focused on the decisions made in practice, the role and usefulness of research in nursing practice.

Interviews and discussion were recorded on minidisk and fully transcribed verbatim.

Field notes were also taken for using in the analysis process.

### Ethical considerations

The local research ethics committee approved the project. Before each interview and focus group discussion, the participants were informed about the purpose of the study, how it would be carried out, and that their participation was voluntary. Participants were advised that they could leave the interviews at any time. Confidentiality would be provided. To ensure confidentiality no names were used. Written informed consent was gained from all participants. All data has remained anonymous.

### Trustworthiness

A number of procedures in data collection and analysis were used to foster 'trustworthiness' [24]. Participants received copies of their interview transcripts for comment and possible revision. No one elected to make changes or to cancel any information. Credibility was established by a team-based approach to analyzing data. Team-based coding, indicating excellent levels of agreement/disagreements in interpretation were resolved through discussion.

### Data analysis

Immediately following each single interview and focus group, the researcher made some notes of what she supposed the key issues arising through the debate were.

In this study, the analysis process proceeded as follows: the transcribed interviews and focus groups were initially read and re-read several times expansively to obtain a comprehensive view of the data, understand the main issues and experiences, and get a good sense of the entire discussion in focus group as well.

Texts were then read line by line, separating passages in to sections concerning similar content. The text was divided into smaller units of analysis that could be reviewed. Meaningful statements and paragraphs were identified and underlined as the unit of analysis. A code was assigned to each meaningful statement and paragraph. Codes were freely generated. Codes and the original files were reviewed by the researcher's director for truthfulness and the participants for precision. Codes with similar meanings were grouped together. The various codes were compared on the basis of similarities and differences and grouped into categories.

The transcripts were read again to validate the codes and categories. For the purpose of abstraction, the relationships between categories were identified and six major themes come up. The themes are kept in mind and the researcher returns to the data to ensure if the themes really fit the data and refine the themes. Categories and themes were discussed with the second researcher who is an expert in the research field. The researchers used MAXqda2 software for the analysis process.

## Results

The participants were approximately 80% (12) female and 20% (3) male. The age of the participants ranged from 25 to 58 years (mean = 40/37, SD = 7/43). The mean years of work experience was 14/9 (SD = 7.26). Participants have been selected purposefully from nursing wards in the three large educational hospitals in Tehran, capital of Iran. Twelve worked on day shifts and three worked on night shifts, all worked full-time. After being contacted seven other nurses participate actively in the study. Focus group participants' ages ranged between 28 and 50 (median 40) and years of practice as staff nurse ranged between 4 and 25 (median 14). Data analysis resulted in the identification of six Up-and-coming themes.

These are presented in table 1 and the following section explains these themes.

**Table 1:** Main themes emerging from analysis of nurses' perspective on research Utilization

**No**	**Main Themes**
1	Degree of support nurses need to be research users

2	Extent of nurses' knowledge and skill to research utilization

3	Levels of educational training involving research

4	Research mindedness

5	Administration and executive challenges in clinical setting

6	Theory-practice gap

Degree of support that nurses need to be research users:

The need for support in emotional, informative and practical areas was found essential.

Participants expressed that there is limited professional and organizational support for conducting and developing nursing research. They further highlighted that the Iranian health care system has not paid enough attention to nurses' support requirements to be research users. Lack of support in providing facilities and equipment, lack of access to online information and as a result a lack of easy access to research reports are some of the major obstructions that were slowing down the use of research in practice.

*In our wards, we do not have a library. Some of our wards have libraries. We do not carry research journals in them (participant 5)*. Similarly another participant describes insufficient facilities.

We don't have any access to the internet, so accessing articles becomes difficult for us. It needs to be on the wards. It needs to be at no cost. I think there needs to be much more on hand. It needs to be much more available.(Participant 8)

Across focus group and single interviews, participants felt that there is no emotional, informational and practical supportive environment for the Iranian nurses. Head nurse (*participant *1) said: *There is little opportunity for our nurses to use research in their practice. They are not provided with enough emotional and training support*.

The nurses frequently pointed out that organizational and motivation supports are the way forward to strengthening individual and organizational development.

### Extent of nurses' knowledge and skill to research utilization

Review of literature revealed a knowledge and skill deficit in conduction research and using it [25,11]. This aspect of the study was explored with the participants of the focus group. They all mentioned that they don't have necessary knowledge and skills toward research utilization. The following explanation was offered by staff nurse: *It is not an easy task for us to base the nursing on research findings; we need the appropriate education to recognize the individual stages necessary. I have not been given this appropriate training in either nursing school or in my years of working as a nurse (Participant 14)*.

The lack of presence of up to date scientific nursing resources was criticized by the participants. *The majority of the journals we do have are not up-to-date. Some of them are not research-based and they are mostly simply newsletters. We do not have any access to any English nursing research journal (Participant 6.)*

All participants in single interviews suggested that some research skills such as data collection and analysis are necessary for the nurses. The majority of participants believed that extensive research skills are required for research utilization.

### Levels of educational training involving research

A number of studies recommended that the level of educational training has a direct impact on nurses' knowledge of research and their attitudes towards it [19,25]. All of the participants in this study believed that the content of research courses and nursing educational resources are not sufficient for teaching and learning of research utilization. They mentioned that there is not a consistency between conducting research and using the research results. On of the participant indicated that: *During my Master of Science training, I only became familiar with research methodology. However I was not taught how to use it in my training (Participant 9)*.

The participant noted that lack of educational facilities, lack of focus on continuing and in service education in research utilization and poor access to expert collogues are the reasons for insufficient research education in nursing. Participants reported on shortcoming of their organization in providing a suitable educational setting.

Participants responded by informing: *We do not receive the appropriate training to utilize research in our training (Participant 11)*.

I have been working as a nurse for 14 years now and I have only been using the same basic training I was taught in nursing school; research is not included (participant 3).

In general the participants in both focus group and single interviews concluded that education of research utilization process is one of the basic and important principles for providing research-based care. They also believed efficient research education leads to research-based practice.

### Research mindedness

Research mindedness was another theme identified from data. Focus groups and single interviews' participants claimed tradition and routine based practice impede provision of research based care and leads to stagnant nursing practice.

In general, the clinical decision-making in Iranian nursing is based on the ward's routine and doctor's orders. Therefore, this means that we are not up to date in daily practice (Participant 7).

Task-centered practice reinforces traditional based practice and this further segregates the nurses from research based practice. As a result nurses don't priorities and focus on acquiring research utilization skills.

The head nurse tells me what to do, whether to apply the dressing to a patient or give drugs. Therefore, I do not actively think for my caring for the individual patient. Nurses do as they are told in their daily practice without thinking about it. Therefore due to this, high quality caring is not available to our patients (Participant 15).

Rushing through responsibilities, task-centered practice and conforming to superiors, all leads to nurses caring for the wards more than the patients. The gradual accustoming of nurses to routine and manual task further reinforces the lack of specialized and skilled mentality.

It is a common opinion that nursing is not a specialty profession such as medicine and therefore it neither does nor needs the use of research (Participant 2).

The study considers that the imbalanced emphasis given by nurses to research conduction and research utilization devalues the importance of research transmission. In addition, research is not considered to be a valuable and useful tool in nursing practice.

Research is only important for nursing educators. Clinical nurses themselves are not involved in research conduction or its utilization. They think it is boring mostly.

(Participant 8)

Despite the emphasis on research as one of the basic and important nursing roles, focus group participants highlighted lack of research role in Iranian nursing care leads to lack of research utilization.

In nursing education there is an emphasis on nurse's research role. However, this is only presented in nursing books and not in the everyday practice of nurses (Participant 4).

Participants have concluded the nurses who do not consider research roles and do not have research thoughts have not been doing research based practice. Therefore, the traditional-based practice has been sustained strongly. Study participants highlighted the need for creating a research culture in nursing practice as a prerequisite for promoting research-based, clinical practice. Additionally, the creation of an appropriate research context could strengthen research mindedness in nursing administrators and staff. They emphasized the culture of the unit to be focused on research utilization.

### Administration and executive challenges in clinical setting

Policy-making and planning, evaluation policy, scientific and research-based management patterns are three sub-themes identified from analysis of the up to date data from this theme.

*Policy-making and planning*: In both focus group and single interview, examples were offered of policy-making and planning:

As research utilization is neither a major priority nor a policy in either nursing education or practice, there are no plans to introduce research utilization into nursing and internalize it in their practice (participant 7 focus group).

Nursing managers/administrators, such as supervisors and head nurses do not see research utilization as a priority in the hospital and ward management (participant 6 focus group).

*Evaluation policy*: The evaluation criteria do not seem effective as the results of research are not utilized in the evaluation process. Insufficient evaluation policy could discourage clinical nurses from research-based practice. According to the participants' claim research utilization is not included in the nursing evaluation checklist.

I am assessed only in my patient notes, rather than in my research-based caring for the patients. (Participant 3)

The participants of focus group discussion also mentioned that if research utilization is not incorporated in evaluation policy, nurses' motivation will decrease in using the research. A single interviewee identified: *Research utilization is not involved in our assessment criteria. Therefore, It neither does nor makes a difference in our evaluation whether we make use of research or not (participant 9). *Inadequate evaluation could sustain traditional-based practice and the nurses imitating their superiors.

*Scientific and research-based management patterns: *The participants highlighted a lack of research-based organizational management in nursing. They further claimed that managers are not serious about research-based nursing.

The approach of nursing managers/administrators does not reflect the perspective that research utilization is of high importance to them. The relationship between the nursing managers and the nursing staff in a clinical setting is not based on science (participant 1 focus group). Our clinical practice relies considerably more on routine and ritual, while

It should be acceptable to our supervisors and head nurses than on research evidence (participant 4 focus group)

In the participants' point of view, the lack of scientific administration models led to insufficient management. These participants explained that they care for patients based on their administrative interests. Participants' experiences indicated that there is a gap between research-based administration and traditional administration. They also mentioned that administrators do not support evidence based practice. As a result, the participants felt that the necessity of suitable planning and policy making with the purpose of introducing high quality and evidence-based nursing care are important. It is worth mentioning that administrators could develop a scientific curriculum and policy to guide nurses in critical thinking and using research results in their practice.

### Theory-practice gap

The gap between research and practice has also become a subject of discussion in nursing. It is believed that the root of many problems in nursing is the wide gap between theory and practice in Iran. Participants also claimed that this ample hole leads to a lack of proper context in research usage.

From analysis of the data from this theme, the subsequent sub-themes were recognized:

Structural/organizational gap

Relationship/affiliation gap

Performance/Behavioral gap

*Structural/organizational gap*: participants believed the Structural gap between education and clinical practice in nursing could reduce the speed of research utilization. They also claimed the there is not consistency between education and practice. So these two structures are not able to meet their mutual needs.

Education and clinical practice in nursing are two different duties as the people in charge of each sector are different. (Participant 10)

*Relationship/affiliation gap*: inconsistency in structural gap leads to relationship/gap and subsequently professional and scientific relationships between academic and staff nurses have been damaged. Participants pointed out the lack of relationship development among different groups of nurses:

When a nursing student is studying at nursing school, hospital and the practice sector is unaware of the students. In the same way, when the same student is practicing nursing his/her university is no longer responsible for that student (Participant 12)

Participants in the focus groups felt less professional relationship between board member and faculties in nursing as two important nursing organizations in Iranian health care system is one of the major reasons for theory-practice gap. They strongly announced that if the professional relationship develops, professional interaction will be improved in nursing.

It is necessary for the board of nursing and the faculties of nursing at the various nursing school to have some sort of communication as the relationship between the two is important (participant 6).

*Performance/Behavioral gap: *The participants viewed the performance gap between education and practice in nursing has a hindering impact on research utilization.

Examples offered by some participants in the single interviews did point to different performances between academic and staff nurses.

In Iran, the majority of PhD students and nursing educators are not very involved with the clinical nursing ward (participant 13).

In both focus groups and face to face interviews, participants had a detailed dialogue about poor professional relationships among nurse-researcher and clinical nurses. This matter has been discussed from different perspectives. They believed a lack of consistency between education and practice in nursing discipline is the result of the lack of resources and facilities, motivation, routine and ritual practice and undifferentiated in nursing responsibilities.

In nursing education, the most recent up to date research findings are available to students. However, when they enter the clinical nursing ward, the appropriate facilities to use the up to date research information are not available. It is for this reason that it is difficult for nurses to carry over the information learned in school into the clinical nursing ward (participant focus group3).

In terms of participants' point of view, closing the gap between academic setting and clinical setting is the outcome of sympathy among these professional groups and it is an important context for research utilization.

### Limitations of the study

Like all studies, this work has some limitations. The results have indicated some potential influencing factors that may be worth exploring with a larger, more representative group of nurses. A key consideration in this study is that since this study has been conducted by qualitative approach, it is not possible to generalize these findings to nursing as a whole.

## Discussion

Findings revealed that a range of different and multifaceted barriers affect the research utilization process negatively. Implementing research evidence involves many influencing factors and is often challenging [26,27].

### Support

According to the results, nurses received least support in the utilization of research findings from ward managers. They also claimed the infrastructure to support research efforts is not established and emotional-informative, practical support was nurses' need to be research active. From the managerial point of view, the most imperative source of support is the ward manager, so it is quite distressing to notice that many nurses felt that they received only a little support from this person.

Lack of support from head nurses is also an important finding in that the development of nursing is a key element of the head nurse's role, not only in providing support and in encouragement on the ward, but also in an attempt to help the use of research results. Rycroft-Malone et al (2004) proposed that a supportive context or environment and adequate facilitation are needed to achieve research-based practice [28]. Nevertheless, Rogers (1994) found that perceived support, in general, was not associated with research utilization, but that support of the director of nursing, the unit director and the chairperson was significantly correlated with research utilization [29]. In particular, senior staff/policy should make certain that the necessary structural and practical support has been recognized and will be supplied. Thus, nurse administrators and other organizational managers and policymakers need to provide health professionals and, in particular, nurses a supportive professional environment to speed research-based practice movement. The role of administrative support and lack of such support in the utilization of research results has been highlighted in numerous previous studies [30,31].

### Knowledge and skill

Extent of nurses' knowledge, skill and levels of educational training involving research utilization were two themes that extract from data and have a close interrelation with each other.

Majority of participants also recognized that they lacked the skills and knowledge necessary to make use the evidence. In general, the participants viewed their research skills as being basic. They believed that many Iranian nurses lack knowledge of the research process. From the participants" suggestions, it seems that the possession of research knowledge may be an important factor for enhancing their skill to evaluate and use research findings. These findings are supported by previous studies [19,17].

Knowledge and skill in research use are needed to build nurses' professional portfolios and gain recognition as science-based providers. If nursing care decisions are to be based on science, infrastructure, skills, and abilities are required of nurses to interpret, use, and conduct research. With this knowledge and these skills come power to change practice and benefit patients [32]. Among the reasons, participants claim that they did not have any educational opportunities and time within the workplace to read and discuss research that is relevant to their daily practice. Inadequate educational preparation in research is also supported by other authors [20,33].

### Educational training

The participants recognized that they needed further education support to improve the quality of nursing care delivered. Authors believe education is seen as one of the main factors underpinning changes and research training is a key way, in which academic departments can increase research capability and capacity [34-36]. In addition, Iranian educational system for nurses should be reviewed for consideration of course content that focuses on using research in practice. At the individual BSc level, nurses need to prepare themselves with the skills needed to evaluate current findings, establish if they are applicable for their setting, and apply these with confidence. However, training research courses should also be made available to practicing nurses.

Despite providing nurse education in Iran at an academic level, research is often seen as a lower priority in nursing practice and research utilization is not well and used in Iranian nursing practice [8].

### Research mindedness

Despite providing nursing education in Iran at the academic level, research evidence and its application to improvements in patient care are neither well understood nor applied in the practice setting. Moreover, participants claimed that Iranian nurses have little motivation and interest to use research results and they do not regard using research as part of their job. The majority of Iranian clinical nurses strongly agree with the statements 'research is not relevant to the everyday work in nursing [8]. This necessarily means that they thought it had no worth in the nursing setting. The patients were seen as work units and the aim of care was to get through the work as quick as possible. This was thought to suppress any ideas of creative or innovative practice. The circumstances of nursing in Iran defined by low to medium salaries, lack of organizational advocacy, nurses' professional rights, and control over professional credential. Nursing roles in Iran are also quite focused on practical matters, consisting mainly of the provision of direct care to patients based on traditional approach instead of research-based practice. Additionally, Iranian nurses work under extensive time pressure. The definite shortage of nursing staff creates an environment of very low nursing autonomy; the delivery of patient care usually falls under medical command. The traditional nature of nursing, in contrast to the scientific position of medicine, appears to delay nursing movement in research. It has also emerged in earlier studies.

The lack of a belief in the value of nursing research, traditional and experience - based practice, unfamiliarity with research reports, rushing through responsibilities, task - centered practice and conforming to superiors leads nurses to be mindless of research.

This in turn strengthens experience and routine-based care. According to Hutchinson and Johnston, depending on experience-based practice threatens nursing and conduct of nursing practice in this manner is 'the antithesis of professionalism[11]. The majority of Iranian nurses are still not convinced of the importance of research to nursing practice and to the nursing profession as well [8]. It is also a barrier to autonomy, and a detriment to quality care.

However, participants believed research mindedness creates innovation in nursing practice; they also claimed that constructing a research friendly culture through appropriate infrastructure promoting the use of research in practice. Meijers et al (2006) reported a statistically significant relationship between research climate, an environment where research use is encouraged and recognized, and research utilization [37].

The delivery of research-based practice is a complex issue that involves personal commitment including a positive attitude, and willingness to keep up to date. So it is essential to change cultural attitudes, create a research culture and building an optimal research environment [38-41,36] and generally creating an atmosphere that is conducive to research activity [40]. Such strategies are to be effective if coherent visions and missions develop in nursing organization to address successfully the challenges outlined above [34].

The nursing organizational values and priorities have played an important role in affecting the ability to develop research capacity. These organizations should formulate policy focus on implementing research, becoming research-based practice as an central part of the nursing strategic plan [42], teaching research use process as part of core duties in nursing universities and, reshaping teaching curricula [38]. Convergences of policy and professional agendas are also facilitating to develop nursing research use [43]. Yet where nurses perceived that a rule existed, they were more likely to be using research-based caring in practice.

### Administration and executive challenges in clinical setting

Participants claim that research use is not a personal responsibility and they address relevant barriers related to lack of managerial commitment to getting research into practice. Lack of organization's support had been experienced by some nurses as contributing to thwarting their efforts at practice innovation. Managers are not concerned with developing professional care. Nurses in this and other studies [11,12] have repeatedly cited a lack of management support as problematic. The healthcare system in Iran does not also provide the incentive for nurses to engage in research or to seek research findings [1]. The findings demonstrate that organizations' political and contextual agenda, organizational structures and procedures have a possible influencing factor on adoption of research findings.

Rogers (1995) claimed that in many cases an individual cannot implement new ideas before the organization has formally adopted them [44]. Moreover, in the current study the nurses reported that research implementation has a strong interdisciplinary component and involves collaboration between individuals and organizations as Parahoo and McCaughen (2001); and Walsh (1997) mentioned in their studies [31,45]. Those nurses who reported support from their nurse managers were also willing to implement research findings in practice [14,46].

Participants believed if research use capability is part of the professional expectation for nurses, then nurses must be forced to learn the new skills, and organizations will need to facilitate required knowledge and skills. The provision of well-defined organizational processes and pathways would assist nurses in promoting research-based change in their practice. To affect research-based practice change, providing a framework of support, sympathetic infrastructures, development of a national strategy to use nursing research, adequately clarified expectations along with encouragement seems necessary [47]. Clinical nurses themselves need to dynamically embrace research-based practice and look for opportunities within organizations to become involved in research opportunities to both support their professional paradigm and development patient care.

Organizations need to be open, have decentralized decision-making processes and imbue a management style that is facilitative rather than 'ordering' [21].

In modern health care, much stress is located on 'transformational leadership'. Transformational leaders create a culture that recognizes everybody as a leader of something. Transformational leaders require rationality, motivational skills, empathy and self-confidence in public presentation [48]. Thus, it is implied that the transformational leader can alter the prevailing organizational culture and create a context that is more conducive to the integration of evidence and practice. Interventions related to work patterns; clinical supervision, communication systems, management structure, quality improvement, and staff development are key determinants to achieve research - based practice.

### Theory-practice gap

According to the results, the research practice gap is still a major force for research-based practice in Iranian nursing and nursing administrators should consider it. Participants believe Iranian nursing should find a way to close the research practice gap. Participants state that nurses with a PhD are not being adequately involved as research-active practitioners. In Iran, the PhD is rapidly becoming the essential degree for faculty positions in nursing universities. This is certainly occurring in many universities around the developing and developed country [49]. As a result, cooperation between academic and clinical staff is one of the main elements driving the movement for research-based care. Some authors confirm the finding that collaborative exchange between service and academia is essential and there is obviously a real need for increased collaboration between researchers and clinical nurses willing to promote the use of research [42,50,51]. The academic staff could act as change agents and play roles of facilitators on nursing research team and assist staff nurses to develop ways to implement the research study findings. Active dialogue between the researcher and the nurse was advisable in order to promote research dissemination. This strategy has been confirm by previously [32,52]. In addition, lack of synergy between two main groups in nursing lead to weakness profession.

In the Iranian nursing situation, the clinical and academic settings are governed by different administrative structures and different visions for research utilization. It seems that mutual interest of promoting research utilization and establishing committees with leaders of both settings are crucial. Clinicians needed to be equal partners in the research endeavor and faculty had to be willing to be flexible and adaptive to clinical realities [42]. Research utilization is a reflective process found both at the individual and institutional levels and it needed interdisciplinary interaction. Collaboration, interaction and recognizing mutual need could lead to narrow research-practice gap.

## Conclusion

The findings reported in this study emphasize that despite Iranian nurses' willingness and desire for research implementation, research utilization appears to be highly complicated, incorporating issues such as administration and executive challenges, lack of support, the wide gap between theory and practice, lack of knowledge and educational training continue to hinder this process. It is worth mentioning that overall, organizational issues were more highlighted in Iranian setting.

These results can have consequences for organizations, nurses, and educators. There is a need for hospital administrators to implement educational programs for research utilization. Moreover, professional organizations have an important role to play in supporting inclusion of research-based care in their policy and evaluation. Research centers developed in each clinical setting would contribute considerably in familiarizing nurses with the value and process of research utilization.

Researchers believe that an international exchange of ideas and practice can yield new knowledge for nursing practice. By exchanging new knowledge, either the new knowledge can be introduced fully to make the practices more efficient and sensitive to the patient, or the new knowledge can be adapted to make it workable in that society's context. In short, nursing in Iran is still a long way from the achieving research that influences practice [8]. However, identifying barriers is just the first step to changing the management of research-based practice.

## Competing interests

The authors declare that they have no competing interests.

## Authors' contributions

MS is Corresponding author. MS and NM were responsible for defining the research questions, Study design, and analysis. MS was responsible for the drafting of the study proposal. NM was responsible for data collection, and the drafting of this paper, which is a summary of her doctoral dissertation. MS read and approved the final version.

## Pre-publication history

The pre-publication history for this paper can be accessed here:


